# Effect of Pirfenidone on Risk of Pulmonary Fibrosis in COVID-19 Patients Experiencing Cytokine Storm

**DOI:** 10.3390/healthcare10122387

**Published:** 2022-11-28

**Authors:** Marian S. Boshra, Ahmed E. Abou Warda, Mahmoud Abdulbasser Sayed, Mohammed H. Elkomy, Nasser H. Alotaibi, Marwa Mohsen, Rania M. Sarhan

**Affiliations:** 1 Clinical Pharmacy Department, Faculty of Pharmacy, Beni-Suef University, Beni-Suef P.O. Box 62514, Egypt; 2 Clinical Pharmacy Department, Faculty of Pharmacy, October 6 University, Giza P.O. Box 12585, Egypt; 3 Teacher’s Hospital, Cairo P.O. Box 11561, Egypt; 4 Department of Pharmaceutics, College of Pharmacy, Jouf University, Sakaka 72341, Saudi Arabia; 5 Department of Clinical Pharmacy, College of Pharmacy, Jouf University, Sakaka 72341, Saudi Arabia

**Keywords:** pirfenidone, lung fibrosis, cytokine storm, mortality, COVID-19, hepatotoxicity

## Abstract

Objectives: Severe stages of COVID-19 infection have been associated with the excessive discharge of pro-inflammatory mediators such as cytokines, resulting in lung deterioration, which progresses rapidly to lung fibrosis leading to acute respiratory distress syndrome. In this investigation, the efficacy and safety of the novel antifibrotic and anti-inflammatory agent, Pirfenidone, were assessed in COVID-19 patients with pulmonary fibrosis secondary to cytokine storm. In this randomized controlled study, we assigned 100 adult COVID-19 patients cytokine storm and admitted to the intensive care isolation unit into either pirfenidone added to the standard therapy (n = 47), or the standard protocol only (n = 53). High-resolution computed tomography of the chest was performed in all patients to evaluate fibrotic lesions and their progression. The results showed that the percentage of patients who developed pulmonary fibrosis during cytokine storm onset in the pirfenidone group relative to the standard group was 29.8% and 35.8%, respectively, with no significant difference between the two groups; while there was a significant increase in the proportion of patients discharged from the isolation unit with pulmonary fibrosis without progression in fibrotic lesions in the pirfenidone group compared to the standard group (21.3% and 5.7%, respectively). Furthermore, there was a significant difference concerning liver enzyme elevation and GIT disturbance incidences in the studied groups (*p* = 0.006 and 0.01, respectively). Our findings show that Pirfenidone inhibits fibrosis advancement in COVID-19 patients with pulmonary fibrosis and is associated with hepatotoxicity and GI distress. It may be beneficial in patients with mild to moderate COVID-19-induced pulmonary fibrosis; however, additional research is necessary.

## 1. Introduction

From December 2019, the spreading of COVID-19 infection became a disturbing event in the whole world [1], and the global death count from post-COVID-19 infection continues to rise, particularly from cardiac injury, kidney failure, and consolidation of ground-glass opacities in specific regions of the lung. Excessive discharge of pro-inflammatory mediators like cytokines results in lung deterioration, which progresses rapidly to acute respiratory distress syndrome (ARDS) leading to lung fibrosis [2,3]. Many studies have shown that ARDS is one of the most important leading causes of mortality in COVID-19 patients [4,5,6]. 

Hyperinflammation, which is almost certainly caused by the cytokine storm, is among the severe symptoms developed by the majority of COVID-19 patients [7]. Numerous studies define hyperinflammation as an extreme form of inflammation accompanied by cytokine storm [8,9]. Cytokine storm syndrome is an umbrella term for clinical syndromes in which hyperinflammation and multi-organ disease result from excessive cytokine release due to uncontrolled immune activation [10]. Excessive local pro-inflammatory cytokine release is thought to be the source of pathological alterations and clinical symptoms of ARDS. In the lung tissue, viral cytopathic-like alterations, infiltration of inflammatory cells, and the presence of viral particles are the chief pathogenic symptoms. Consequently, substantial lung injury in COVID-19 patients is attributed to both direct viral infection and immunological hyperactivation [11].

Lung fibrosis, which is associated with the inflammatory response, occurs as a secondary event that induces acute diffuse damage to the alveoli, hypoxia, and edema [12]. 

Additionally, the generation of pro-inflammatory cytokines (cytokine storm) is considered a critical event that paves the way to morbidity and mortality in severe COVID-19 patients [13]. The mechanism linking hyperinflammation to fibrosis involves a protein expressed by the macrophages, and epithelial and alveolar cells in the lungs, known as galectin-3 (gal3). This is a carbohydrate-binding protein whose pleiotropic functions deserve therapeutic attention for their roles in the inflammatory response, lung fibrosis, and hypoxia [14]. In COVID-19-afflicted individuals with severe disease, proliferating T cells appear to overexpress gal3. Additionally, some macrophages express several markers linked with fibrotic processes, including, for example, TREM2 and SPP1 [15,16].

Antifibrotic medicines may be beneficial in preventing the cascade of significant or fatal consequences resulting from severe COVID-19 infection [17]. Antifibrotic and anti-inflammatory actions are found together in the drug pirfenidone, which suggests that the dual action of pirfenidone may result in additional clinical benefits, such as tolerability at lower doses and less toxicity [18]. Pirfenidone was approved in 2011 by the US Food and Drug Administration (FDA) and the European Medicines Agency (EMA) as a first-line therapy for varying-severity Idiopathic Pulmonary Fibrosis (IPF) [19]. The different effects of pirfenidone are based on several mechanisms, as it can inhibit apoptosis, and regulate tumor necrosis factor-alpha (TNF-α) and -beta secretions, transforming growth factor-5 (TGF-5) activity, and cellular oxidation [20]. Taking into account these mechanisms and the well-investigated pathophysiology associated with COVID-19 infection, it seems logical to anticipate that pirfenidone and other antifibrotic therapies could be of value in managing COVID-19-induced lung fibrosis and in protecting pneumocytes and different cells from the invasion of COVID-19 and the cytokine storm [21,22].

This study was designed to analyze the proportion of patients who survived lung fibrosis among COVID-19 patients receiving pirfenidone alongside standard treatments, as well as to monitor any pirfenidone-related adverse events.

## 2. Patients and Methods

### 2.1. Study Design

The present study was an open-label randomized controlled trial that included 100 adult patients with severe COVID-19 infection with cytokine storm syndrome admitted to the intensive care unit of the isolation department of Teachers Hospital, Cairo, Egypt, between 30 June 2020 and 30 November 2021. 

The first group of the studied COVID-19 patients (pirfenidone group) (n = 47) received pirfenidone treatment in addition to the standard management of the Egyptian Ministry of Health COVID-19 protocols, while the second group (Standard group) (n = 53) received only standard management. 

### 2.2. Protocol and Study Population

An Institutional protocol for COVID-19 standard of care was applied to all patients as a background treatment. The aforementioned protocol consisted of antiviral therapy such as remdesivir 200 mg LD then 100 mg once daily as a maintenance dose, or lopinavir/ritonavir 400/100 mg twice daily, or ivermectin 36 mg every three days, anti-inflammatory drugs such as dexamethasone 6 mg once daily, tocilizumab 400 mg, or infliximab 200 mg as a single dose, and anti-coagulation prophylaxis with enoxaparin administered subcutaneously once a day if D-dimmer was between 0.5–1.0 ng/dL, or enoxaparin administered subcutaneously twice daily if D-dimmer was >1.0 ng/dL.

Throughout the hyperinflammation phase, patients were randomly assigned in a 1:1 allocation to receive either the standard treatment or pirfenidone along with the standard treatment at a dose of 600 mg per day in the first week of treatment, then 1200 mg per day in the next week, then 1800 mg per day in the third week. This was done by simple randomization using a computer-generated table of random numbers.

Patients were included in the study if they met the following inclusion criteria: 

(A) Adult patients more than 18 years old with COVID-19 infection confirmed with polymerase chain reaction (PCR) test. 

(B) Only severe COVID-19 patients admitted to hospital or transferred to the intensive care unit after the COVID-19 viremic phase (7–10 days) with systematic hyperinflammation (cytokine storm) recognized as C-reactive protein (CRP) ≥ 100 mg/L (normal values < 6 mg/L) or ferritin ≥ 900 ng/mL (normal value < 400 ng/mL), in the existence of lactate dehydrogenase (LDH) > 220 U/L, and at least one of the features: partial pressure of arterial oxygen to fraction of inspired oxygen ratio (PaO_2_/FiO_2_) < 300, blood oxygen saturation ≤ 93% on room air (RA), respiratory frequency ≥ 30/min, or a decline in lung involvement, recognized as an increased number and/or extension of consolidated pulmonary areas, increased demand for FiO_2_ to maintain stable O_2_ saturation, or worsened O_2_ saturation of >3% with stable FiO_2_.

Patients were excluded from the study if: 

(A) The patient was known to suffer hypersensitivity to pirfenidone. 

(B) The patient was diagnosed or suspected of idiopathic pulmonary fibrosis before the diagnosis of COVID-19. 

(C) Pirfenidone was absolutely contraindicated (e.g., in liver cirrhosis or elevation of liver transaminase levels). 

(D) The patient was pregnant or lactating.

### 2.3. Patient Follow-Up and Clinical Outcomes

Patient demographics and clinical data, including comorbidities, current medications oxygenation, and pulmonary function parameters, such as oxygen saturation (SaO2), forced vital capacity (FVC), forced expiratory volume (FEV1), and PaO_2_/FiO_2_ ratio, were collected upon admission. All patients recruited in the study were screened for developing pulmonary fibrosis after the onset of cytokine storm in each group.

**Figure 1:**
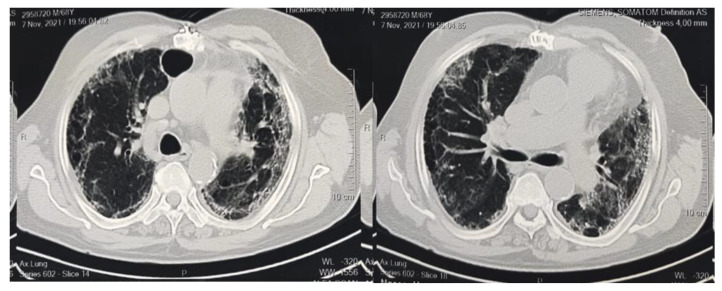
CT imaging indicates bilateral marked pulmonary infection with diffuse ground glass opacities, areas of air trapping, and pulmonary thickened interstitium with subsequent architectural distortion, basal bronchiectasis, and honeycombing appearance.

Chest high-resolution computed tomography (HRCT) was performed every two days from the time of hyperinflammation for the purpose of diagnosing lung fibrosis as shown in Figure 1. HRCT-defined pulmonary fibrosis was any distribution other than the subpleural/basal, extensive ground-glass opacity (extension > reticular abnormality), numerous micronodules (bilateral, primarily upper lobes), discrete cysts (multiple, bilateral, apart from honeycombing areas), diffuse mosaic attenuation/air trapping (bilateral, in three or more lobes), or bronchopulmonary segment/lobe consolidation [23]. 

The CT images were independently assessed by two senior thoracic radiologists. Visual assessment for fibrotic lesions involved in the lung was assigned for each CT scan [24]. The lungs were evaluated in a total of six different regions, with the top regions located above the carina, the intermediate regions between the carina and the inferior pulmonary veins, and the bottom regions under the inferior pulmonary veins [25,26]. The abnormality was estimated to the nearest 10% of the lung parenchyma. By averaging the six different lung regions, it was able to determine the overall percentage of lung involvement [27] utilizing a scale from 0 to 5, with 0 denoting no engagement, 1 denoting less than 5% involvement, 2 denoting 5–25% involvement, 3 denoting 26–49% involvement, 4 denoting 50–75% involvement, and 5 denoting more than 75% involvement [28,29].

The primary outcomes of the study were the proportion of patients in each group who developed lung fibrosis, which was indicated through CT scans during the duration of the cytokine storm, and the percentage of patients with pulmonary fibrosis who were discharged from the isolation unit without significantly progressing the condition of fibrosis defined as no increase in fibrotic lesions in the lung parenchyma and no development of acute respiratory distress syndrome. The secondary outcomes included the amount of time it took for patients to recover from cytokine storms without developing pulmonary fibrosis, the length of hospital stays (LOS), overall mortality, and any side effects of pirfenidone such as hepatotoxicity and GIT disturbances.

### 2.4. Statistical Analysis

#### 2.4.1. Sample Size Calculation

Based on a previous trial [30], A minimum sample size of n = 92 patients was indicated for the current study based on 90% power to detect an effect size f2 of 0.704, assuming a two-sided tail hypothesis and an alpha-level = 0.05. Considering a possible loss of about 10% of patients, the sample size was increased to 100 patients.

#### 2.4.2. Descriptive and Inferential Statistics

The Shapiro–Wilk test was employed to test for the normality of the quantitative variables. Mean and standard deviation were utilized to represent normally distributed quantitative variables, while median and interquartile ranges were utilized for non-normally distributed quantitative variables for qualitative data; frequency and percentages were used. The independent *t*-test and Mann–Whitney U test were employed for normally and non-normally distributed quantitative data, respectively. Chi-square and Fisher’s exact tests were the tests for qualitative data. The duration needed to discharge from the isolation unit and duration of hospitalization (in days) between the two groups were compared using time-to-event analysis, and statistical significance was tested using the log-rank test. The statistical tests employed in this study were two-tailed and a statistically significant difference was considered at a *p*-value < 0.05. IBM SPSS software version 20.0 (Armonk, NY, USA: IBM Corp) was employed to perform all statistical analyses. 

### 2.5. Ethics Statement 

The study was carried out after approval from the Research Ethical Committee of the Faculty of Pharmacy, Beni-Suef University, approval serial number (REC-H-PhBSU-21021). The study was performed according to the good clinical practices recommended by the Declaration of Helsinki and its amendments. Informed written consent was obtained from each study participant.

## 3. Results

### 3.1. Patient Demographics 

**Figure 2:**
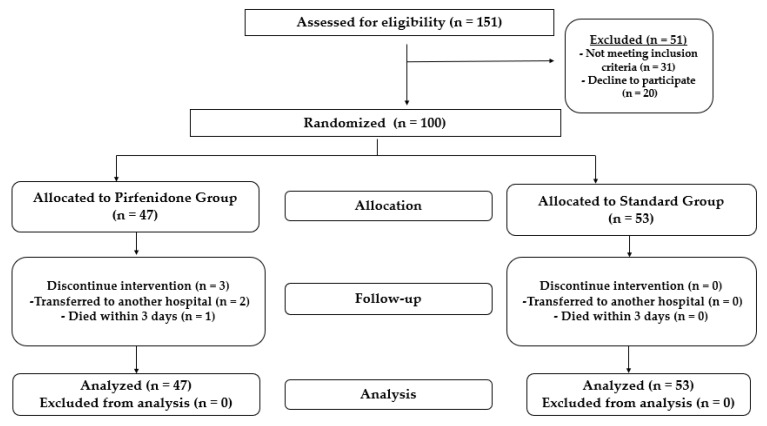
Flow chart showing the study design and patient randomization.

A total of 151 patients were assessed for eligibility; 51 were excluded from the study as 31 did not meet the inclusion criteria and 20 declined to participate. Using intention-to-treat analysis (ITT), 100 patients (68 males) were randomized into two groups unless they withdrew consent. The first group (53 patients) received the standard treatment protocol, and the second group (47 patients) received the standard treatment protocol plus pirfenidone, as illustrated in Figure 2. Six patients withdrew informed consent prior to the initiation of therapy in the pirfenidone group. 

The mean age of the standard group and pirfenidone group was 62.42 ± 11.77 and 64.68 ± 10.60 respectively (*p*= 0.317). Most patients presented with many comorbidities. The most common comorbidities identified in the standard group and pirfenidone group in percentage were hypertension (58.5% versus 44.7%), diabetes (39.6% versus 29.8%), ischemic heart disease (28.3% versus 10.6%), atrial fibrillation (9.4%versus 8.5%), and asthma (5.7% versus 6.4%), respectively. Table 1 summarizes the baseline characteristics of all the patients.

### 3.2. Patient Clinical Features, Baseline Pulmonary Functions, and Inflammatory Markers

The levels of inflammatory markers LDH, ferritin, and CRP were similar (*p* > 0.05) between the two groups. The two groups exhibited a significant difference only in the level of D-dimer, which was higher in the pirfenidone group (2.75 ± 10.86) versus 0.857 ± 1.32 for the standard group, (*p* = 0.013). Regarding pulmonary functions, there was no significant difference in pulmonary functions between the two studied groups including FVC, FEV1, and Po_2_/Fio_2_ ratio. There was also no significant difference in oxygen saturation, or need for high or low oxygen, between the two groups. In addition, WBCs, neutrophil/lymphocyte ratio, and liver enzymes recorded similar values (*p* > 0.05) in the two groups. Table 2 summarizes the baseline inflammatory markers of the recruited patients following cytokine storm. The pulmonary functions and clinical features of all patients are presented in Table 1.

### 3.3. Clinical Outcomes and Follow-Up

The outcomes were based on, firstly, the CT imaging that was utilized to identify the prevalence of pulmonary fibrosis in the patients. The broncho vascular bundle distortion, traction bronchiectasis, fibrotic strips, architectural distortion, and interlobar septal thickening were the CT imaging characteristics that indicated pulmonary fibrosis. The frequencies of each CT finding were compared between the two groups and found to have no significant difference, as illustrated in Table 3. There was no discernible difference between the standard group and the pirfenidone group in terms of the percentage of patients who developed pulmonary fibrosis during the cytokine storm period. The percentage of patients who developed pulmonary fibrosis was 35.8% in the standard group and 29.8% in the pirfenidone group. 

The number of patients who were released from the isolation unit with pulmonary fibrosis but no progression in lung fibrotic lesions was 3 in the standard group and 10 in the pirfenidone group, respectively, indicating a significant difference between the two groups (*p* = 0.034). In addition, the changes in the percentage of pulmonary fibrosis involvement on CT scans from initial findings to time to discharge in both groups are illustrated in Table 4. There was a significant difference between the two groups at the time of discharge at scale 4 at *p* = 0.011 and scale 5 at *p* = 0.009. 

Second, when comparing the two groups, there was no significant difference (*p* > 0.05) in the average number of days needed for recovery from cytokine storm without pulmonary fibrosis. These numbers were 4.66 ± 2.56 days in the standard group and 4.60 ± 2.93 days in the pirfenidone group. Both groups received the same amount of treatment. 

**Figure 3:**
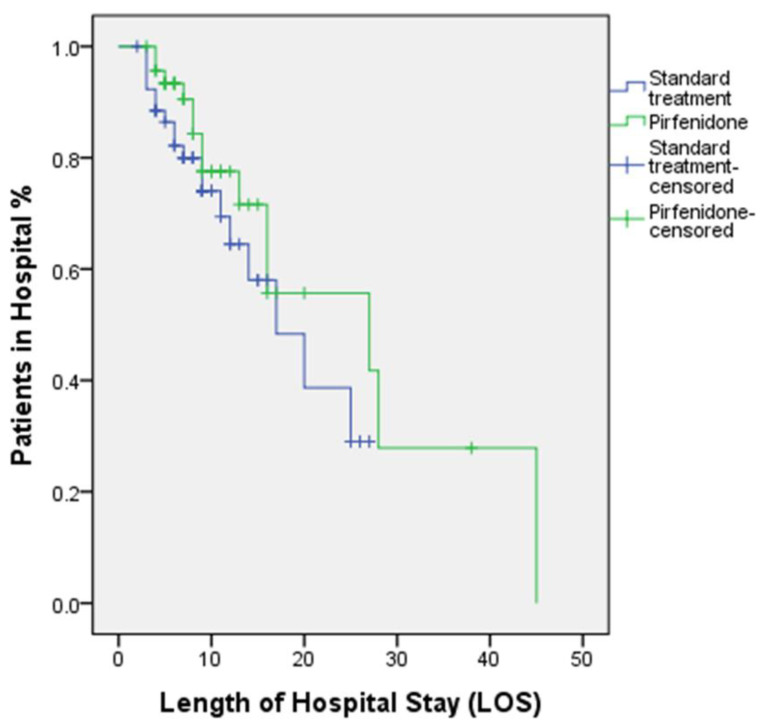
Kaplan–Meier curves and log-rank test show the time needed to discharge from hospital *p* = 0.256.

Within the isolation unit, the percentage of patients who died of pulmonary fibrosis was 30.2% in the standard group and 21.3% in the pirfenidone group, which was also non-significant (*p* > 0.05). Furthermore, the duration of stay in the isolation unit, LOS, and overall mortality rate recorded in the two groups failed to demonstrate significant differences (*p* > 0.05). Figure 3 depicts the Kaplan–Meier curves and log-rank test for comparison of the time needed to discharge from the hospital.

At the same time, the incidence of elevation of liver enzymes (i.e., when the upper limit of the normal range was exceeded by three or more times) in the studied groups was 15.1% in the standard group versus 40.4% in the pirfenidone group, signaling a significant difference (*p* = 0.006). However, this difference was without clinically relevant repercussions as all elevations encountered with aminotransferase were reversible. Also, GIT disturbances (nausea, vomiting, diarrhea, decreased appetite, and constipation) in the pirfenidone group were significantly recognized (36.2%) when contrasted with the standard group (13.2%) (*p* = 0.01). Table 5 summarizes the clinical outcomes and follow-up in both groups.

## 4. Discussion

Patients of COVID-19 die as a result of a cytokine storm, acute inflammation, and oxidative stress, which lead to ARDS and multi-organ failure [2]. Therefore, it is possible that anti-inflammatory and antifibrotic therapies such as pirfenidone might have clear benefits in counteracting COVID-19-induced cascade and post-COVID-19 fibrosis progression in the acute phase of the illness. In this study, the influence of pirfenidone on the incidence rate of pulmonary fibrosis in COVID-19 patients during the cytokine storm phase within the context of SARS-CoV-2-related acute illness was investigated. 

The principal finding of this research was that the pirfenidone group, compared with the standard group, exhibited a significant reduction in the proportion of patients discharged from the isolation unit without a progression in pulmonary fibrosis. Although not significant, a lower incidence of pulmonary fibrosis and associated death rate in the isolation unit were observed among patients who received pirfenidone vs. standard therapy. These findings corroborate and expand the findings of the CAPACITY trial (study 004), which showed that pirfenidone increased progression-free survival by 36%, while also reducing the probability of mortality or disease advancement by 36% [31].

Progressive pulmonary fibrosis is a cause of death for some patients with SARS-CoV-2 respiratory distress syndrome. Some studies showed that pulmonary fibrosis is more common in people with prolonged viral infections, suggesting that antifibrotic medication should be delivered early to be more clinically effective. Some treated COVID-19 patients have radiological and physiological anomalies associated with fibrotic lung disease; medium-long-term antifibrotic medication may be needed to speed the entire healing process [32,33].

Based on the fact that fibrosis and hyperinflammation are both associated with COVID-19-related bilateral interstitial pneumonia, antifibrotic agents such as pirfenidone can be a valuable aid in preventing serious or fatal complications from COVID-19 in patients with ongoing infection, or in those who have recovered from infection but still have residual fibrotic lung lesions. Antifibrotic activity start time is a crucial factor to think about. It is possible that a treatment regimen combining antifibrotics and immunomodulants might lead to a crucial and much more potent pharmacological synergism. This is because the evolution towards a severe pulmonary fibrotic lung state can be very rapid in some severe cases of SARS-CoV-2, which can prevent antifibrotic therapy from acting in time [17,32].

Lung damage with extensive fibrosis and quick development of RDS are hallmarks of the most severe stages of COVID-19 infection, which are characterized by a sudden and overwhelming release of proinflammatory mediators. The fact that over-inflammation and cytokine storm are linked to a poor prognosis highlights the significance of early recognition of cytokine storm and the execution of anti-inflammatory treatment. Avoiding irreversible ARDS and the multi-organ dysfunction associated with COVID-19 requires prompt management of the hyper-inflammatory response [34,35].

Another encouraging trend in favor of pirfenidone was investigated in the Acat M. et al. study that was conducted on hospitalized patients diagnosed with severe COVID-19 pneumonia, as it was discovered that pirfenidone can prevent the development of fibrosis. In addition, it can aid in dose reduction and/or non-use strategies for methylprednisolone therapy, which is associated with numerous adverse effects [36]. These observations were similar to the conclusions of a study that was carried out on patients with severe COVID-19 infection, and indicated that beginning pirfenidone medication as soon as possible is associated with a better treatment and survival outcome compared to corticosteroids. This is especially true for patients who are at higher risk, such as the elderly and those who have comorbidities [30].

Furthermore, another study on pirfenidone was reported in a pooled cohort from phase 3 trials where the reduced risk of IPF-related and treatment-emergent IPF-related death over time was noticed [37].

In comparison to standard medication, pirfenidone had no meaningful effect on the length of isolation unit stay, LOS, or overall mortality rate. In patients with pulmonary fibrosis following SARS-CoV-2 infections, there is no clear evidence so far evaluating the efficacy of pirfenidone in reducing all-cause mortality. These mortality analyses need to be conducted over longer follow-up periods and for a larger sample size per arm. In addition, the presence of comorbid diseases can substantially impact survival in patients suffering from IPF [38,39].

As the hallmark of a cytokine storm is the release of proinflammatory cytokines, it was mandatory to quantify the levels of CRP, LDH, ferritin, as well as neutrophil to lymphocyte ratios, in either the pirfenidone or the standard group. No significant difference was observed regarding these biomarkers providing no added value for pirfenidone over the standard anti-inflammatory therapy.

Furthermore, the patients who received pirfenidone experienced an unexpected elevation in D-Dimer level compared to the standard group. This finding showed a discrepancy with the previous studies, which reported the potential of pirfenidone in reducing D-Dimer in patients with IPF confirming the role of pirfenidone in the downregulation of inflammatory pathways during cytokine storm [40].

However, there is growing evidence that COVID-19 patients are at high risk of developing acute pulmonary embolism; therefore individuals with severe COVID-19 may need to receive anticoagulant medications to improve outcomes [40]. Therefore, further research would be of great interest in this area to find out the exact role of pirfenidone in coagulation pathways.

Regarding the safety profile of pirfenidone, the drug was associated with a significant rise in liver enzymes compared to standard therapy. Following pirfenidone exposure, glutathione/N-acetylcysteine (GSH/NAC) conjugates were discovered in microsomal/primary hepatocyte incubations. The fact that these conjugates are produced from pirfenidone explains why the drug has a deleterious effect on the liver. Additionally, rats fed with pirfenidone had GSH/NAC conjugates in their bile and urine. These conjugates are consistent with the synthesis of a quinone methide intermediate. Two possible paths may have led to the formation of the intermediate, direct dehydrogenation of pirfenidone to the quinone methide intermediate, and oxidization of pirfenidone to 5-hydroxymethyl pirfenidone and then sulfation to form a benzyl alcohol-sulfate derivative. These findings contribute to our comprehension of how idiosyncratic toxicity is caused by pirfenidone [41] and further corroborate the current guideline on pirfenidone product labels in Europe and Canada. According to these guidelines, for the first six months of commencing therapy with pirfenidone, tests to assess liver function are recommended every month and at three-month intervals thereafter [42].

Moreover, gastrointestinal disturbances, such as nausea, vomiting, diarrhea, decreased appetite, and constipation were more prevalent in the pirfenidone group compared to the control group. All results were consistent with those of previous studies [17,43,44].

The present study had some limitations that should be taken into consideration, such as the short duration of patient follow-up, which may necessitate additional research to more clearly demonstrate the long-term antifibrotic effect of pirfenidone to lessen the severity of fibrosis, as well as the incidence of pulmonary fibrosis in COVID-19 patients and the extent of its recovery. Concerning this issue, there is a need for additional large-series as well as randomized controlled investigations.

## 5. Conclusions

Pirfenidone does not prevent the development of pulmonary fibrosis during acute sickness, but it does slow the development of fibrotic lesions induced by the cytokine storm in individuals with COVID-19. Pirfenidone is associated with hepatotoxicity and GI distress. Future research should focus on demonstrating the long-term efficacy of pirfenidone in reversing the course of mild to moderate post-COVID-19 pulmonary fibrosis.

## Figures and Tables

**Table 1:** Baseline and Demographic Data for Recruited Patients in Both Groups.

Parameters	Pirfenidone Group (n = 47)	Standard Group (n = 53)	*p*
Age (years), Mean ± SD	64.68 ± 10.60	62.42 ± 11.77	0.317
Gender (male), n%	32 (68.1%)	36 (67.9%)	0.986
BMI (kg/m^2^), Mean ± SD	29.07 ± 3.77	29.06 ± 4.24	0.892
Temperature °C, Mean ± SD	38.0 ± 0.85	38.1 ± 0.93	0.794
Patients need low-flow oxygen, n%	12 (25.5%)	19 (35.8%)	0.287
Patients need high-flow oxygen, n%	31 (66.0%)	34 (64.2%)	1.000
Pulmonary functions			
Oxygen saturation, Mean ± SD	81.09 ± 12.44	81.45 ± 12.59	0.561
FVC %, Mean ± SD	75.96 ± 12.18	75.87 ± 12.23	0.758
FEV1 %, Mean ± SD	71.77 ± 11.42	71.85 ± 11.77	0.742
Po_2_/Fio_2_ ratio	141.6 ± 73.89	152.5 ± 71.35	0.499
Comorbidities			
Hypertension, n%	21 (44.7%)	31 (58.5%)	0.229
Diabetes, n%	14 (29.8%)	21 (39.6%)	0.401
Ischemic heart disease, n%	5 (10.6%)	15 (28.3%)	0.044 *
AF, n%	4 (8.5%)	5 (9.4%)	1.000
Asthma, n%	3 (6.4%)	3 (5.7%)	1.000
Others, n%	12 (25.5%)	13 (24.5%)	1.000
Current smoking habits, n%	9 (19.1%)	17 (32.1%)	0.174
Medications			
Remdesivir, n%	46 (97.9%)	46 (86.8%)	0.063
Lopinavir/Ritonavir, n%	16 (34.0%)	11 (20.8%)	0.177
Ivermectin, n%	5 (10.6%)	13 (24.5%)	0.116
Infliximab, n%	18 (38.3%)	21 (39.6%)	1.000
Tocilizumab, n%	40 (85.1%)	43 (81.1%)	0.790

*p*: *p*-value for comparing the studied groups *: Statistically significant at *p* ≤ 0.05.

**Table 2:** Baseline inflammatory markers of recruited patients following cytokine storm in both groups.

Parameters	Pirfenidone Group (n = 47)	Standard Group (n = 53)	*p*
D-Dimer, Mean ± SD	2.75 ± 10.86	0.857 ± 1.32	0.013
LDH, Mean ± SD	447.1 ± 242.2	483.1 ± 291.4	0.824
Ferritin, Mean ± SD	1158.1 ± 962.2	866.16 ± 763.9	0.096
CRP, Mean ± SD	133.59 ± 90.3	127.4 ± 90.45	0.671
WBCs, Median (IQR)	8.7 (7.0–12.4)	7.3 (4.8–11.2)	0.058
Neutrophil/lymphocytes ratio, Mean ± SD	10.85 ± 7.47	9.21 ± 7.09	0.268
ALT, Mean ± SD	48.93 ± 34.68	43.56 ± 25.4	0.842
AST, Mean ± SD	48.65 ± 29.95	50.78 ± 35.4	0.968

*p*: *p*-value for comparing the studied groups.

**Table 3:** Initial CT findings indicating pulmonary fibrosis features following cytokine storm in both groups.

Parameters	Pirfenidone Group (n = 47)	Standard Group (n = 53)	*p*
Broncho vascular bundle distortion, n%	25 (53.1%)	19 (35.8%)	0.749
Traction bronchiectasis, n%	37 (78.7%)	31 (58.4%)	0.451
Fibrotic strips, n%	7 (14.8%)	4 (7.5%)	0.529
Architectural distortion, n%	29 (61.7%)	22 (41.5%)	0.532
Interlobar septal thickening, n%	10 (21.2%)	12 (22.6%)	0.422
Honeycombing appearance, n%	8 (17.02%)	15 (28.3%)	0.167

*p*: *p*-value for comparing the studied groups.

**Table 4:** Comparison between changes in percentage of pulmonary fibrosis involvement on CT scans from initial findings to time to discharge in both groups.

Scale	Initial Lung Involvement			Lung Involvement at Time of Discharge		
	Pirfenidone Group (n = 47)	Standard Group (n = 53)	*p*	Pirfenidone Group (n = 47)	Standard Group (n = 53)	*p*
Scale 1, n% Less than 5% involvement	11 (23.4%)	12 (22.6%)	0.558	7 (14.9%)	9 (17.0%)	0.140
Scale 2, n% 5–25% involvement	24 (51.1%)	26 (49.1%)	0.500	15 (31.9%)	17 (32.1%)	0.138
Scale 3, n% 26–49% involvement	7 (14.9%)	7 (13.2%)	0.517	9 (19.1%)	7 (13.2%)	0.296
Scale 4, n% 50–75% involvement	5 (10.6%)	8 (15.1%)	0.360	11 (23.4%)	3 (5.7%)	0.011 *
Scale 5, n% More than 75% involvement	-	-	–	5 (10.6%)	17(32.1%)	0.009 *

*p*: *p*-value for comparing the studied groups, *: Statistically significant at *p* ≤ 0.05.

**Table 5:** Clinical Outcomes and follow-up in both groups.

Parameters	Pirfenidone Group (n = 47)	Standard Group (n = 53)	*p*
Development of pulmonary fibrosis, n%	14 (29.8%)	19 (35.8%)	0.532
Mortality within isolation unit, n%	10 (21.3%)	16 (30.2%)	0.365
Discharge without progression in lung fibrosis, n%	10 (21.3%)	3 (5.7%)	0.034 *
Time to recovery without lung fibrosis (days), Mean ± SD	4.60 ± 2.93	4.66 ± 2.56	0.734
Duration of cytokine storm (days), Median (IQR)	3 (3–6)	4 (3–6)	0.596
Isolation unit stay (days), Mean ± SD	9.34 ± 5.21	9.11 ± 4.99	0.909
Length of hospital stay (LOS) (days), Mean ± SD	11.23 ± 8.48	9.72 ± 6.03	0.448
30-day mortality, n%	14 (29.8%)	18 (34.0%)	0.674
Incidence of hepatotoxicity, n%	19 (40.4%)	8 (15.1%)	0.006 *
ALT, Median (IQR)	80 (52–182)	53 (38–79)	0.030 *
AST, Median (IQR)	60 (33.75–82)	41 (29–65)	0.102
Incidence of GIT disturbances, n%	17 (36.2%)	7 (13.2%)	0.01 *

*p*: *p*-value for comparing the studied groups, *: Statistically significant at *p* ≤ 0.05.
